# Upper Limb Physical Rehabilitation Using Serious Videogames and Motion Capture Systems: A Systematic Review

**DOI:** 10.3390/s20215989

**Published:** 2020-10-22

**Authors:** Andrea Catherine Alarcón-Aldana, Mauro Callejas-Cuervo, Antonio Padilha Lanari Bo

**Affiliations:** 1 Software Research Group, Universidad Pedagógica y Tecnológica de Colombia, Tunja 150002, Colombia; 2 School of Computer Science, Universidad Pedagógica y Tecnológica de Colombia, Tunja 150002, Colombia; mauro.callejas@uptc.edu.co; 3 School of Information Technology and Electrical Engineering, University of Queensland, Brisbane 4072, Australia; antonio.plb@uq.edu.au

**Keywords:** serious videogames, motion capture, upper limbs, physical rehabilitation, telerehabilitation, inertial sensors, inertial measurement unit (IMU), state of the art

## Abstract

The use of videogames and motion capture systems in rehabilitation contributes to the recovery of the patient. This systematic review aimed to explore the works related to these technologies. The PRISMA method (Preferred Reporting Items for Systematic reviews and Meta-Analyses) was used to search the databases Scopus, PubMed, IEEE Xplore, and Web of Science, taking into consideration four aspects: physical rehabilitation, the use of videogames, motion capture technologies, and upper limb rehabilitation. The literature selection was limited to open access works published between 2015 and 2020, obtaining 19 articles that met the inclusion criteria. The works reported the use of inertial measurement units (37%), a Kinect sensor (48%), and other technologies (15%). It was identified that 26% used commercial products, while 74% were developed independently. Another finding was that 47% of the works focus on post-stroke motor recovery. Finally, diverse studies sought to support physical rehabilitation using motion capture systems incorporating inertial units, which offer precision and accessibility at a low cost. There is a clear need to continue generating proposals that confront the challenges of rehabilitation with technologies which offer precision and healthcare coverage, and which, additionally, integrate elements that foster the patient’s motivation and participation.

## 1. Introduction

One of the sustainable development objectives suggested by the United Nations (UN) is oriented toward the universal and integral coverage of health services, and the reduction of its inequalities, in order for everyone to be in good health [1]. In accordance with the above, it is taken into account that inequalities contribute to millions of people with disabilities facing difficulties in carrying out their basic daily activities. This is more pronounced among people from communities with fewer opportunities and resources, which are generally geographically located in areas that are distant from the services required for rehabilitation processes [2].

Of the different types of disabilities, motor disability is considered to be one of the main limitations to human beings carrying out their basic activities, affecting the quality of life of the individual, as well as that of those around them [3]. In the last few years, telemedicine and telerehabilitation have been strengthened with the implementation of diverse technologies that support rehabilitation processes, oriented toward providing patients with the services required, reducing the number of journeys to main cities, where, in general, specialists, hospitals, clinics, and centers equipped with the technology for the therapies are located. The benefits of telemedicine are more evident in cases associated with traveling and the mobility of the patient, costs, or other factors, for instance, in a situation of isolation or confinement such as that experienced worldwide due to COVID 19, which does not allow people to travel somewhere that is adapted for the necessary therapy session for the patients’ recovery [4].

Although in the last few years there have been many technological proposals that support physical rehabilitation, there are still difficulties and gaps in the area which represent an opportunity to contribute to improvements in biomechanical data capture accuracy, the coverage and affordability of health services, and the flexibility and motivation offered to the patients.

With the purpose of identifying the advances and the options available, in order to contribute to the improvement of motor rehabilitation processes, this review includes works published between 2015 and June 2020, oriented toward the support of upper limb physical rehabilitation, which use videogames and a motion capture system. These publications mainly show the use of the Kinect sensor and inertial sensors as motion capture systems. At the same time, it is identified that the works included mainly support motor rehabilitation in people who have suffered a stroke, and another aspect that stands out is the use of commercial systems on the market, which offer different videogames for motor rehabilitation. The objective of this systematic review is to determine the main contributions to this type of rehabilitation in order to identify the opportunities and challenges that should be taken into consideration in future proposals, focused on the improvement in quality of life of people with motor disabilities.

## 2. Materials and Methods

This section provides a description of the process and criteria taken into account to conduct the article selection included in this documental research, according to aspects of the PRISMA method (Preferred Reporting Items for Systematic reviews and Meta-Analyses) [5]. This allowed the authors to critically identify, select, and evaluate the relevant research, as well as compile and analyze the data from the studies included in the review.

### 2.1. Eligibility Criteria

The eligibility criteria taken into consideration for inclusion of the studies in this review were (i) that they were published in English, (ii) that they were published within the last 5 years, in the period 2015–June 2020, (iii) that the full text was open access, and (iv) that the type of document was an article, systematic review, state-of-the-art review, or journal.

Concerning the second aspect, the period mentioned was selected, given that as, from 2010, when Kinect was created, and until 2015, its use became popular in different contexts. After 2015, it is noticeable that there was an upsurge of companies and projects using other motion capture systems and integrating serious videogames, in addition to the Kinect sensor, in the field of rehabilitation, which is the main interest of the present study. Another relevant element in this review is that the studies included had therapeutic purposes of rehabilitation or telerehabilitation of the upper limb using videogames and some motion capture system, regardless of the gender and age of the population which participated in the validation of the proposals described.

### 2.2. Search Strategy

The search of the publications was carried out in four academic databases: Scopus, PubMed, IEEE Xplore, and Web of Science. The following search terms, classified into four groups, were used: (i) medical aspect: rehabilitation, health, “physical therapy”, musculoskeletal, telerehabilitation, “tele-rehabilitation”, “tele rehabilitation”; (ii) use of videogames: videogames, “video games”, video-games, “serious videogames”, “serious games”, “serious video games”, exergames, exergaming, “active videogames”; (iii) motion capture system technology: “inertial sensor”, “motion capture”, mocap, “motion capture system”, wearable; iv) segment or part of the body the rehabilitation is focused on: “upper limb”, elbow, shoulder, arm, wrist, humerus. In the search parameters used in the databases (see Table 1), in each group, the operator OR was included between the different terms considered to be synonyms, and, to separate the groups, the operator AND was used, thereby enabling the search to include at least one relevant term from each group in the data consultation.

The terminology used to refer to motion capture technology often changes between scientific domains. For instance, in clinical studies, it may be possible that focus was given to the manufacturer name. In other papers, alternative terms may have been used, such as simply “accelerometers” or “motion sensing”. We recognize this is a limitation of the methodology adopted in this paper, which may have prevented some papers from being listed in the first stage.

### 2.3. Description of the Selection Process of the Study

The selection process of the works related to the review topic included four phases: firstly, the identification of the studies, in which all the records that respond to the search parameters in each database were taken into account; secondly, the application of a filter, using the eligibility criteria, in order to select the works related to the purpose of the review, which are available and can be accessed; thirdly, a “screening” phase, which filtered out works, eliminating those that did not adjust to the focus of the investigation and/or those which appeared in multiple databases; finally, an inclusion phase, allowing for the identification of documents to be part of the detailed analysis of the systematic review.

## 3. Results

This section shows the findings of the selection process of the study, as well as the characteristics of the works included in the analysis and the individual results presented in those publications.

### 3.1. Selection of the Study

**Figure 1:**
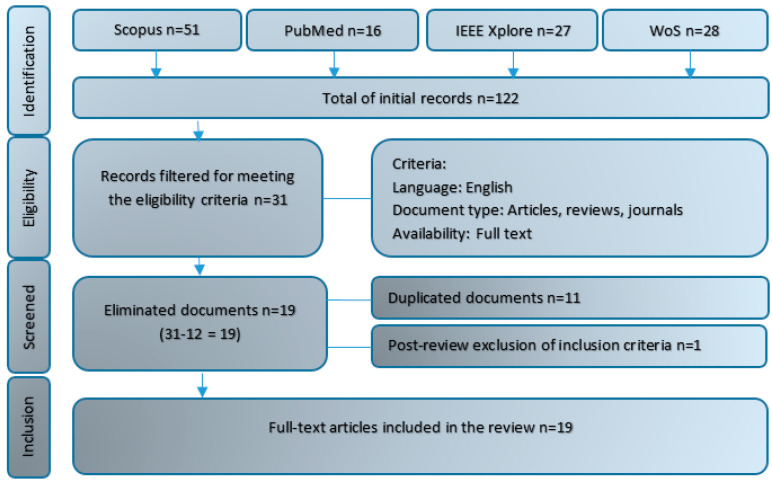
Systematic process used in the selection of articles, based on PRISMA (Preferred Reporting Items for Systematic reviews and Meta-Analyses).

Figure 1 presents the systematic process for the selection of peer-reviewed articles, in which it was identified that a total of 122 documents, published between 2015 and June 2020 and which included the search terms, were found on the databases. In essence, they are studies that focused on the support of upper limb physical rehabilitation, with the use of videogames and motion capture systems. From the total, after applying the eligibility criteria described, 31 works were left; afterward, 11 were eliminated as they were duplicated, and one referred to a book of abstracts from a conference [6]. Thus, the number was reduced to 19 documents, which were directly related to the topic of this review.

### 3.2. General Characteristics of the Study

The main characteristics of the 19 works included in the review could be classified into four groups: (i) according to the motion capture system used; (ii) according to the diagnoses or clinical condition the investigation focuses on; (iii) population included in the validation process; (iv) availability (affordability) of the technology used (motion capture system, videogame, technological platform) in the investigation.

#### 3.2.1. Motion Capture Systems Reported in the Studies

**Figure 2:**
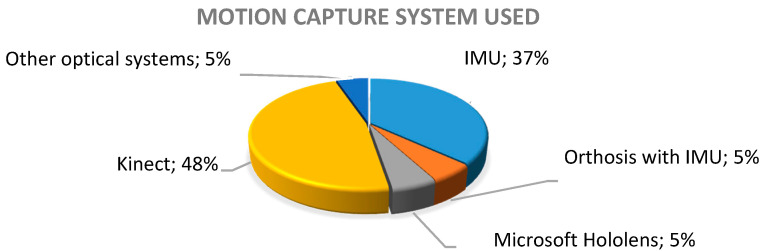
Percentage of the use of motion capture systems.

Regarding the motion capture systems reported to be used in the 19 studies, nine (48%) used Microsoft Kinect, seven (37%) used inertial measurement units (IMUs), one (5%) used a passive orthosis (which integrates inertial sensors, which would add up to 42% for the use of inertial sensors in these works), one (5%) of the studies used Microsoft HoloLens, and the remaining 5% corresponded to a study which reported a systematic review in a period different from that established and, therefore, it was not considered in the review (see Figure 2).

#### 3.2.2. Diagnosis or Clinical Condition on Which the Technology Described in the Works Was Focused

**Figure 3:**
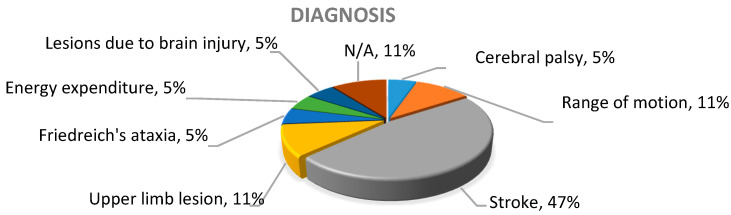
Diagnoses in the works analyzed.

In the studies analyzed, it was identified that 47% of the investigations focused on the treatment of people who suffered a stroke, 11% addressed situations related to the range of movement (ROM), another 11% contributed to the treatment of any injury in the upper limb, 5% oriented their investigation toward people with Friedreich’s ataxia, 5% focused on the treatment of children with cerebral palsy, 5% analyzed energy expenditure in the execution of physical activity, and 16% did not focus on an illness or clinical condition in particular, but on the analysis of technology; thus, they were classified as “not applicable” (N/A), as observed in Figure 3.

#### 3.2.3. Population Involved in the Validation of the Results

**Figure 4:**
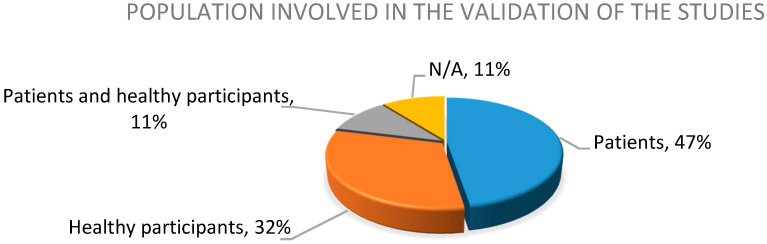
Percentage of the population involved in the study.

In the validation process (Figure 4), 47% of the studies involved patients (with a 21.66 median and a 19.77 standard deviation), 32% validated their proposal only with healthy participants, 11% made a correlational validation between patients and healthy participants, and the remaining 11% did not validate their proposal with a specific population, given that it had a technical focus (drift correction or systematic review of the literature, mainly).

#### 3.2.4. Affordability of the Technology Used

**Figure 5:**
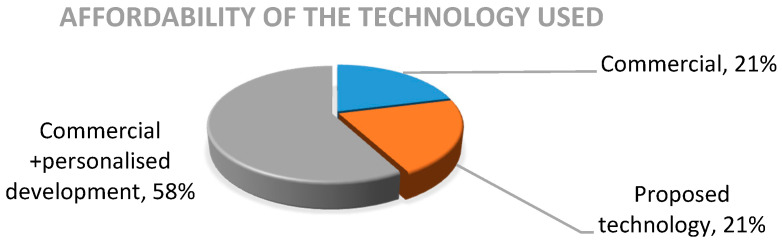
Distribution of the technology used.

With regard to technology availability and affordability, 21% of the works included in this review used commercial products focused on physical rehabilitation. Another 21% proposed systems, referring to the development of technology in academic and/or investigative environments. Most of the studies (58%) were classified as “mixed”, given that the technology used involved a combination of commercial products and some personalized development (mainly videogames), as observed in Figure 5.

**Figure 6:**
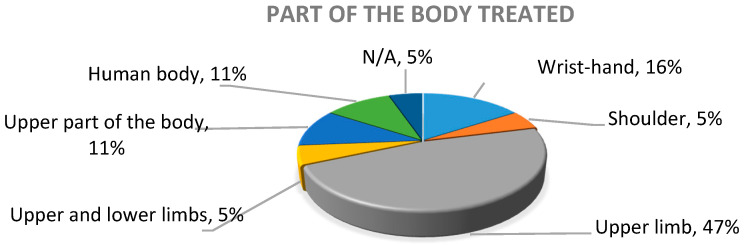
Distribution of the part of the body treated in the study.

An additional aspect in the global analysis of the literature is that, although, within the search parameters, the upper limbs were included, it was identified that there was diversity regarding the part of the body being focused on in the works, as presented in Figure 6. It can be observed that 47% referred, in a general way, to the upper limb, 16% referred specifically to the wrist and the hand, 5% analyzed the range of movement of the shoulder joint, 5% included both upper limbs and lower limbs, 11% oriented the treatment toward the upper part of the body, another 11% focused on the movement of the whole human body, and the remaining 11% had a different focus; thus, they did not analyze any part of the human body.

### 3.3. Technologies as Support in the Physical Rehabilitation of the Upper Limb

Table 2 presents the main characteristics of each of the 19 studies analyzed and identifies how they supported physical rehabilitation using videogames and motion capture systems.

The terms rehabilitation and habilitation, according to the World Health Organization [26], are two processes which “enable persons with disabilities to attain and maintain their maximum independence, full physical, mental, social, and vocational ability, and full inclusion and participation in all aspects of life”. Rehabilitation is defined as the group of methods geared toward the recuperation of an activity or function lost or diminished by a trauma or illness, and it covers a wide variety of activities, including medical care rehabilitation, physiotherapy, psychotherapy, language therapy, occupational therapy, and support services. In this sense, physical rehabilitation is oriented to the recovery of the patient’s motor function by the physical medicine and rehabilitation team.

For the autonomous development of different basic activities, the movement of various parts of the body is required, especially the upper limbs, which allow the realization of diverse complex manual activities [27]. In this sense, in the literature, diverse proposals were found oriented toward processes of upper limb physical rehabilitation, denoting marked trends concerning the use of motion capture systems and videogames.

#### 3.3.1. Use of Motion Capture Systems in Upper Limb Physical Rehabilitation

**Figure 7:**
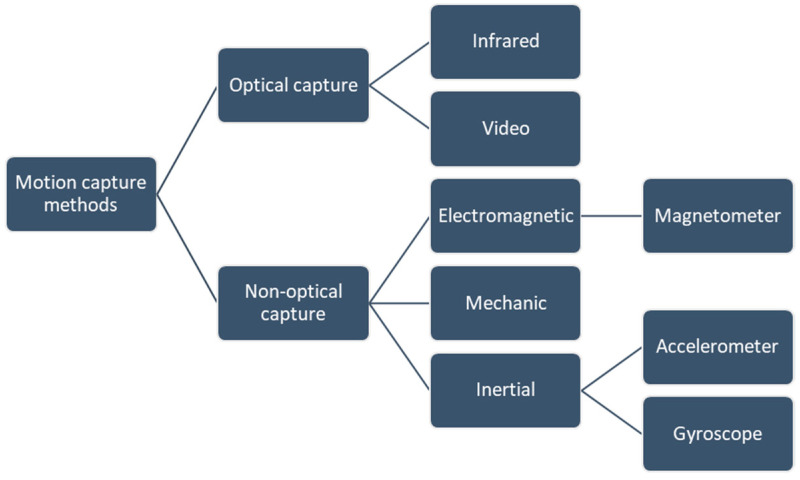
Main motion capture system methods [33].

Motion capture (MOCAP) is the process of acquiring motion by combining software and hardware [28] and is understood as a technique for recording motion and its corresponding transformation into a digital model. It is commonly used in areas such as entertainment, robotics, medicine, and physical rehabilitation, among others [29,30]. Specifically in the field of physical rehabilitation, it is used to identify the effectiveness of appropriate therapy plans [31,32], which when integrated with information and communication technologies in this field, provides therapeutic assistance to patients under the modality of telemedicine. Biomechanical motion capture systems can mainly be optical and non-optical, as shown in Figure 7.

##### Optical Systems Used

Optical systems that use infrared light require the location of markers at specific points on the individual’s body. Then, using a configuration with multiple cameras, properly placed around the capture space, the position of the reflective markers is recorded [34].

The measurement of human movement with optoelectronic systems offers precision due to the position of the retroreflective markers, and that depends, to a great extent, on the optical characteristics of the camera system and the algorithms implemented in the monitoring software [35]. Microsoft Kinect is an example of an optical system for motion capture without markers. This system can detect 25 joints of the human body of six people at the same time and provides precise information on depth data or corresponding original red/green/blue (RGB) data [36].

In this review, most of the works described how they involved a Kinect sensor as an optical system for motion capture in the development of the research. Some used the sensor, and, in addition, they proposed new products to support rehabilitation. For example, [10] evaluated the usability and performance of the KineActiv platform developed in Unity Engine and incorporating Microsoft Kinect V2. Its purpose was to encourage patients to do the rehabilitation exercises prescribed by the specialist, who could control the patient’s performance and correct errors in their execution along the way. In addition, this work included a web platform allowing the physiotherapist to monitor the results of the session, control the patient’s health, and adjust the rehabilitation routines. At the same time, [17] proposed a system denominated GoNet V2, which was associated with the Microsoft Kinect V2 game controller. It was aimed toward physical and rehabilitation specialists, and, through the recording, storage, and management of information, it supported the treatment and evaluation of the range of movement of the joint. In [18], the Kinect sensor and a game developed in a previous project (ICT4Rehab) were used in order to corroborate whether serious videogames could be used as an evaluation tool for the functioning of the upper limbs in the treatment of motor deterioration in patients with Friedreich’s Ataxia, even with a patient sitting in a wheelchair.

Furthermore, [14] determined the spatial precision and the validity of the measurement of Microsoft Kinect V2, using the videogame Mystic Isle, developed as a rehabilitation game. In this case, they compared the results of the sensor with a motion capture system using standard markers, Vicon, which is another optical motion capture system incorporating markers, which uses infrared cameras to track the three-dimensional location of the reflective markers placed on the body. This work presented satisfactory results in the improvement of the motor function and the performance of daily activities in people with a chronic cerebrovascular accident. Regarding the results of the visual comparative analysis with Vicon, for the case of the hand and the elbow, Kinect V1 showed good precision in the calculation of the movement trajectory, but its validity was limited in terms of the movement of the shoulder. For its part, [15] presented five experiments, three of which were application cases, using devices part of the research project called REHABITATION. In one of these cases, a videogame was proposed in addition to the use of Kinect, which fostered the rehabilitation of the upper limbs in stroke patients. In this case, the purpose was the evaluation of the usability perceived. In this aspect, it was ranked as “excellent” on the scale of usability system (SUS) and as “good” on the modified scale of usability system (mSUS).

Other investigations did not focus on the development of new products, but rather on the validation of different attributes in the use of technologies in motor rehabilitation. In [22], the authors evaluated Kinect’s capacity to find movement performance indices through a reliability analysis between sessions and tests. Specifically, reliability was analyzed using eight performance indices: medium velocity, normalized medium velocity, peaks of normalized velocity, logarithm of dimensionless jerk, curvature, spectral arc length, shoulder angle, and elbow angle. In the results of the study, acceptable reliability and sensitivity were mentioned in all the sessions for medium velocity, logarithm of dimensionless jerk, and curvature measured by Kinect for healthy individuals and stroke patients.

In the same way, in [21], the feasibility, efficiency, and safety of the JRS Wave commercial system were evaluated. This software is part of the rehabilitation system called Jintronix (JRS) which was launched by the company Jintronix [37] and uses Microsoft Kinect as its motion capture system. JRS Wave has tasks already set up regarding the upper limb and balance, standing, and walking, and it was used in the rehabilitation of patients hospitalized due to stroke. At the same time, it has a telemedicine system allowing doctors to manage the information of the patients and monitor the physical rehabilitation tasks. The main result referred to the efficiency in the differences of activity levels of the use of rehabilitation technology in comparison to regular rehabilitation. At the same time, in [15], five experiments were described, three of which were cases of application, using devices proposed in the framework of the investigation project developed by the authors. One of these cases, in particular, was related to the aspects addressed in this document, in which the authors proposed a videogame and, along with Microsoft Kinect, fomented the rehabilitation of upper limbs in post-stroke patients.

Another approach identified in the works was that of proposals to optimize the data capture by the Kinect sensor. For example, in [16], a methodology was proposed to extract and evaluate the therapeutic movements of the game-based rehabilitation, executed in environments which were not controlled or supervised. This methodology was oriented toward isolating the relevant movements and eliminating strange movements from the data captured by Kinect, involving the development of computer models that can efficiently process large volumes of data for their later kinematic analysis. Using the Kinect sensor and Microsoft SDK, in [23], three predictive algorithmic models were applied: a Gaussian process regression (GPR), a locally weighted k-nearest-neighbor regression, and linear regression (LR), in order to calculate the mechanical work carried out by the human body and subsequent metabolic energy. The determination of the body segment properties, such as segment mass, length, center-of-mass position, and radius of gyration, were calculated from the Zatsiorsky–Seluyanov’s equations of de Leva, with adjustments made for posture cost. The results showed that the Gaussian process regression slightly outperformed the other two techniques and that it was possible to determine the physical activity energy expenditure during exercise, using the Kinect sensor. Therefore, the estimates for high-energy activities, such as jumps, could be made with accuracy, but not for activities which require low energy such as squats and other activities with stationary positions.

With regard to the use of optical systems, in addition to Kinect, the use of a glove called the 5DT Data Glove Ultra from the company 5DT [38] was presented in the research. This glove was initially designed for computer animation, but it has since been used in other fields. It is fabricated with an elastic material and uses fiber-optic sensors in each of the five fingers to detect changes in the global position of the finger [39]. In the review presented in [24], two documents were included that used this glove as a motion capture system. The first presented the development of a videogame platform with virtual reality that integrated a 5DT Data Glove Ultra and a PlayStation 3 videogame console, for the rehabilitation of adolescents affected by cerebral palsy. This had the purpose of contributing to improving hand movement and the consistency of the bones in the forearm. The other document presented a rehabilitation plan involving videogames, using a PlayStation 3 console and the 5DT Data Glove Ultra for the rehabilitation of the hand of pediatric patients with hemiplegia. In this review, a third document was presented that used an infrared transmitter fixed with a Velcro strap to the hand of the patient and an infrared camera (Nintendo Wiimote) as a motion capture system, which captured the infrared transmission in order to generate an image of the patient in the virtual environment.

On the other hand, in [8], the potential offered by Microsoft HoloLens was explored, i.e., an optical device placed on the head which does not require markers or sensors for the following of the arm or the hand. An application was developed with augmented reality, using the engine from the game Unity and the Microsoft HoloToolkit, for the improvement of the range of movement of the shoulder, allowing a perfect remote interaction with the personal doctor. The work, using the Likert questionnaire, identified good levels of motivation and ergonomics in the proposed technology, from the perspective of a group of patients, as well as that of rehabilitation specialists.

##### Non-Optical Systems Used

Non-optical systems are based on small inertial sensors with built-in accelerometers, gyroscopes, and magnetometers, which allow the recording of data associated with movement in an integrated storage device; these systems are characterized by their low cost, accuracy, and ease of use in ambulatory environments [33]. Portable systems with IMUs are ergonomic, portable, and sensitive, and they can obtain relevant data quickly and accurately in order to make correct decisions related to the intervention of the patient [40].

Different works implemented IMUs due to their potential, such as the case presented in [9], in which a rehabilitation system that integrates videogames and portable technology was proposed, allowing exercises to be realized at home, in order to help the remote recuperation of stroke patients presenting a disability in the upper limbs. The system developed had two principal components: the game engine environment and the software of the therapist to remotely track and follow the progress and achievements of the patients. With regard to the hardware proposed for motion capture, a server in a Raspberry Pi connected wirelessly to a development platform and an MPU6050 sensor was implemented, with a flexible sensor for the detection of flexion and resistance of the fingers and a pulse sensor in order to control the cardiac frequency. Through a survey, the authors identified a great potential for the developed system to facilitate the rehabilitation process of patients from the comfort of their homes and under the remote supervision of the therapist.

Understanding the advantages of the use of inertial sensors, some works focused on an improvement in their efficiency and, taking into account that one of the limitations that IMUs have shown is the problem of drift, in [12], a drift correction method was proposed on the basis of a rest pose magnetometer (RPMC), for the measurement of combined inertia and the following of the arm in real time with a magnetometer. This method corrected drift while the user was relaxing, involving a precalibrated direction of the magnetic field. The commercial system ArmeoSenso was used and a videogame was developed to validate a method following arm movement, resulting in precise monitoring, low latency, and good rhythm, including in environments with proximity to ferromagnetic materials, such as in the home. In the same way, another work optimizing the data generated by IMUs was presented in [11], whose authors began from the premise that classifying a large number of arm movements with IMU-based systems is a difficult task. Therefore, they built a single wrist-mounted device with an inertial sensor and a temperature sensor, to explore the possibility of increasing the classification accuracy of IMU-based systems. The data obtained were pre-processed, and the secondary characteristics were calculated using principal component analysis (PCA) for dimensionality reduction; then, several automated learning models were applied to select the optimal model for speed and accuracy. The results showed that adding a thermal sensor to the IMU-based system significantly increased the classification accuracy in 24 arm movements in healthy participants from 75% to 93.55%.

Another aspect of the research was the accuracy offered by IMUs, which is why they were compared with systems recognized as gold standards in motion analysis [41,42,43,44,45], obtaining acceptable and trustworthy results in different fields, including that of medicine. Moreover, one of the five experiments described in [14] approached the comparison of IMUs to the Vicon Motion Analysis system configured with seven Vicon Bonita infrared cameras. The experiment was conducted on the measurement of the range of movement of the shoulder joint, specifically with three movements: shoulder abduction, external rotation of the shoulder joint, and horizontal adduction. The papers showed the high utility of IMUs in simple monitoring activities, thanks to their ease of connection and handling. Furthermore, the type of system used and the expected period of use influenced motion detection and its characteristics.

Furthermore, [25] took advantage of IMUs in the implementation of a passive orthosis in order to detect movements of the elbow and hand through a classification mechanism, in order to evaluate the progress, or its opposite, in the motor recovery of post-stroke patients, implementing a system that can be used at the patient’s home, demonstrating that the mechanism of adaptation was effective in 78.6% of the sessions, making it appropriate as a self-adjusting tool for machine-based exercise.

Other works used commercial systems involving IMUs, which presented stability in their operation and offerred reliability in the data they provide, allowing the evaluation of their contribution to rehabilitation processes. On the one hand, Bimeo is a sensor-based rehabilitation device aimed at stroke patients and other neurological patients. This device offers a motivating virtual reality environment, which aims to make the therapy effective and motivating for patients and also offers therapists a support tool to monitor and control the Bimeo process [46]. This system was used in [19] to evaluate the short-term effects of competitive and collaborative games in arm rehabilitation. The participants’ subjective experience was quantified using the “intrinsic motivation inventory” questionnaire after each game, and they also used a final questionnaire on game preferences. Exercise intensity was quantified using the Bimeo system, according to wearable inertial sensors that measured hand speed in each game. The results of the work indicated that both competition and cooperation could increase patient motivation to play and that exercise intensity increased when the play partner was a family member or friend.

The ArmeoSenso system [47] involves virtual reality and is based on IMUs for the training of the function of the upper limbs. It also includes therapy software with videogames and automatically evaluates the arm movement [48]. In [20], a feasibility study was carried out on the development of unsupervised arm therapies in self-directed rehabilitation processes carried out in patients’ homes. In this study, after the training given by the specialist, patients with arm hemiparesis used the system in their homes for six weeks with an average duration of 137 min per week, identifying that home therapy is safe and contributes to guiding the rehabilitation process.

The Myo bracelet, developed by Thalmic laboratories, is a portable movement and gesture control device. The newest version of the system, which consists of eight EMG sensors and IMUs, allows the user to control events from a computer (or other device) via a Bluetooth connection, which has been widely used in research environments because of its accessibility [49]. The Myo bracelet is an electromyographic detection device, i.e., the sensors can detect biometric changes in the user’s arm muscles as they move, determining the user’s intentions [50], offering high precision, depending on the location and orientation in which it is used [51]. The bracelet has been used in different contexts, and one of the concerns regarding home interventions is low adherence; thus, [7] evaluated the feasibility of a new intervention that combines a gaming technology integrating evidence-based biofeedback and training strategies. In this case, the purpose was to use the bracelet and videogames in the experiment to identify the recruitment rate of 8–18 year old patients with cerebral palsy and their continuity in home therapy for one month. The Myo bracelet was also used by children with upper limb disabilities in [13] to evaluate a game developed and adapted to be controlled with the bracelet. According to the results, they identified that the participants felt comfortable and were able to interact with the game and, therefore, there was high acceptance due to the fun that was experienced. In this way, the authors reported that the Myo bracelet made it possible to improve accessibility to videogames and improve the exercise of the upper limbs.

#### 3.3.2. Use of Videogames in Upper Limb Physical Rehabilitation

Another aspect of interest in this review was to identify how the use of videogames is being addressed in upper limb physical rehabilitation. It was found that, although some studies involved the use of commercial products, most of them developed new videogames adapted to the needs of the target population of the investigation.

In [7], the commercial videogame “Dashy Square” was used [52], which was launched in 2016 by KasSanity and was adapted so that the participants in the investigation executed therapeutic gestures with their hands to control the actions of the game on the screen; this was used as a motivational environment involving goals to tackle muscle weakness and selective motor control. It was determined that the training focusing on the solution, proposed in this work, in combination with videogames that provide biofeedback, had a positive influence on the activities that require enrolment of participants and practice at home, and that there was more retention of patients during a monthly intervention, which were the parameters defined. Another commercial product used was the videogame therapy software included in the ArmeoSenso system, which has been previously mentioned, which is oriented toward the recovery of the function of the arm. In [12], a therapy game called “Meteors” was implemented in ArmeoSenso. This game involves a virtual robotic arm that coincides with the movement of the arm of the player and is used to catch meteors which fall on a planet. At the same time, in [20], in addition to the videogame “Meteors”, the game “Slingshot” was used, with the purpose of training arm coordination and improving precision in the movements for aiming and extending the arm. In this game, the patient exercises the flexion/extension of the elbow. To this end, the patient holds a virtual slingshot with which they have to shoot stones to set targets which may be stationary or in motion, while the size and velocity may vary. In these games, the score is calculated according to performance. The level of difficulty can be dynamically adjusted in order to maintain motivation and commitment during the recovery of the patient.

In [21], the JRS Wave software was used, which was designed in collaboration with occupational and physical therapists, using criteria of motor relearning. In this system, amusing and attractive videogames were programmed in order to exercise the upper limbs, practice balance, and walking. In this work, although the authors did not provide details about the videogames used, they claimed that they could be adjusted to different levels of complexity and speed, and they determined that these tools increase adherence and joy for exercising, thereby increasing the amount of repetitive exercise carried out by people with limited mobility.

Furthermore, works were identified in which, regardless of whether the motion capture system was commercial or an independent proposal, specific videogames for the development of the investigation were presented. In this group of works, the development of videogames that used the platform Unity was noticeable [53]. For example, in [8], with the purpose of treating any deficit of the upper limb which deteriorates the range of motion, a videogame was designed and developed from traditional rehabilitation exercises with the Rolyan range-of-motion shoulder arc. The game presents a curved tube, with mobile colored rings around it. They have to be moved from one side of the tube to the other, achieving a complete range of motion of the upper limb. This improves motor planning abilities and visual monitoring. The videogame can also be used with the HoloLens glasses using augmented reality. In this case, the user, with the movement of the hand, controls a virtual cursor throughout a predefined virtual trajectory. Furthermore, in [10] using Unity, active videogames were created to be executed with Kinect in the platform KineActiv, through which patients interact with a gamified user interface that implements a personalized game environment for each type of exercise.

Using Unity 3D and the C# programming language, [13] developed and evaluated a videogame involving a jigsaw puzzle with three levels of difficulty, adapted to be used with the Myo bracelet. In the game, the gestures perceived by electromyography such as double touch, shake the hand to the right and the left, close fist, and separate the fingers, were the commands to interact with the videogame and put together the jigsaw puzzle, contributing to motor recovery, as well as to cognitive aspects of the patient. The evaluation tool was based on an evaluation questionnaire of educational games and showed that the videogame was stimulating and attractive, and that it fulfilled the expectations of the patients (5–15 years old children with disabilities). It was also identified that the genres preferred by the children were those of adventure, reasoning, and creativity. In the same way, [14] developed “Mystic Isle” with Unity 3D, a multiplane, full-body rehabilitation videogame which uses Kinect V2 as an input device. This game, depending on the therapeutic treatment, can be used in either a sitting or a standing position, and different movements can be followed: gross motor movements (steps, jumps, squats) or fine motor movements (shake a hand, turn the palm face up, open and close the hand). With this system, the player is tracked in a three-dimensional space and, afterward, the data are registered in real time by the associated software, showing good results related to motor function and the execution of basic daily activities in chronic post-stroke patients.

In one of the cases in [15], a rehabilitation videogame was presented which implements C# and UnityScript. This game is oriented toward the physical recovery of upper limbs in neurological patients, through the interaction with two scenarios related to their daily lives. The first scenario presents a bookshelf, and the player has to avoid the books in it falling. The second scenario simulates a kitchen where the player, in a given time, has to pick up an object requested in a written text. Both games are controlled by the movement of the hands, which is detected by Kinect V2. For that reason, the first time that the game was used, a calibration was carried out in order to guarantee that the patient could reach all corners of the screen with their virtual hands.

The use of Kinect and its utilities was also noticeable in [16], through “Recovery Rapids”, a personalized videogame to be used with Kinect, in which data were captured to evaluate the relevant movements of the continual therapeutic game. In this work, a methodology was presented to isolate the relevant movements and eliminate strange movements from the data obtained through Kinect during the therapeutic game, incorporating the implementation of computational models to efficiently process great volumes of motion capture data compiled in noncontrolled environments.

In [18], a serious videogame called “WipeOut” was used (developed by the authors for a previous project), and, in conjunction with the use of the Kinect sensor, a functional evaluation of the upper limbs was carried out. With the movement of the arm and the position of the hand, the player must wipe the screen to discover an image. The evaluation was carried out contrasting the performance (time and precision) in the execution of the activity in two groups: one of patients with Friedreich’s ataxia and another of healthy individuals.

Using C# and Microsoft XNA game studio, in [22], a game was designed to be used with Kinect, the purpose of which was to be able to monitor the movement of the hands of the participants. For this, the player had to move their hands to intercept and catch several colored balls which went in the direction of the person, according to the guide given by the system. The program registers the positions of the joints of the upper part of the body (hand, wrist, elbow, shoulder, center of the shoulder, position of the head, and waist) to be able to carry out the respective analysis. The results of the study indicated acceptable reliability in the session and between sessions when the Kinect sensor and the proposed game were utilized, which as comparable to the data calculated with robotic systems or clinical evaluation scales.

Another example of videogame development for physical rehabilitation was included in the SCRIPT system [25], designed for wrist and hand rehabilitation, through the execution of daily exercises mediated by three interactive videogame options. In one of these games, the patient must open and close their hand, with this movement controlling a seashell that opens and closes to catch fish. They also used the videogame “Crocco”, in which the subject must move a crocodile on the screen. This game is available in four variants. In the simplest one, the player flexes and extends the wrist to avoid obstacles; in the second case, lateral arm movements are added to move the crocodile laterally on the screen; the third variant of the game requires the grip movement to simulate the crocodile eating fruit, and the fourth variant includes all the previous movements. The third game included in the system described is called “Labyrinth”, in which the patient moves the cursor through a maze, in three variations of the game. In the simplest one, the cursor can be moved up and down with anteroposterior hand movements, as well as to the left and right, encouraging the flexion–extension of the wrist. In the second variation, prone supination of the hand is used to open and close the doors in the labyrinth. The third variation includes a gripping gesture to take a key before opening doors. In this research, although the participants did not practice as much as initially advised, such that great variability in the duration of the sessions was obtained, relative ease in the movement of the wrist compared to the movement of the hand was identified, as well as that the proposed system can be used as an adaptive regulator of the difficulty of the exercise, depending on the performance of each subject. Likewise, in [10], a rehabilitation environment was presented, in which one of its modules corresponded to a videogame engine, which included three games designed specifically for rehabilitation purposes located in the patient’s home. However, in this case, the system allowed the recovery of both the upper and the lower limb, through the use of designed games.

Another approach to the use of developed videogames, specifically for motor recovery, was the comparison of competitive, cooperative, or individual videogames. In this regard, [19] designed four videogames for arm rehabilitation. One of those videogames was competitive, in which the patient plays against another person (a friend, relative, or therapist). There were also two cooperative games, in which the patient and another player play together against the computer; lastly, there was an individual game in which the patient plays alone against the computer. It was identified that competitive games contributed, to a greater extent, to the functional recovery and improvement in the quality of life of the patients in comparison to conventional rehabilitation exercises.

#### 3.3.3. Diagnosis and Treatments Supported by Technology

The publications analyzed in this review were oriented toward the support of physical rehabilitation using technology. Most of these works fostered the recovery of patients who suffered from a particular clinical condition. Below, there is a description of the contribution made by each of them.

##### Technological Support in Post-Stroke Motor Recovery

A cerebrovascular accident, also known as a stroke, is an acute event, caused primarily by a blockage (accumulation of fatty deposits on the inner walls of the blood vessels), which prevents blood from flowing to the brain. They can also be caused by bleeding from a blood vessel in the brain or by blood clots [54]. One of the main effects of a stroke, in patients and their families, is the limitation in carrying out basic daily activities; thus, one of the main purposes of rehabilitation therapies is to improve the movements of the arm and promote the recovery of lost function through rehabilitation therapy [55].

In the review, it was identified that most of the works were geared toward the support of motor rehabilitation treatments in post-stroke patients, as is the case of [9,14,16,19,20,21,22,25], and the five cases presented in [15]. These works described different experiments that involved videogames and motion capture systems in the motor recovery of neurological patients to contribute to the improvement in their quality of life and facilitate the work of the medical staff involved.

##### Technological Support in the Recovery from Other Diagnoses

Another diagnosis mentioned was cerebral palsy, which is the most frequent cause of motor disability in children and the third cause of disorders in neurological development. It is, in essence, a group of nonprogressive disorders which occur during the development of the brain, in the fetal phase or the first years of life. These disorders affect mobility and postural development; therefore, they also make carrying out different activities more difficult [56]. In [7], the feasibility of using technology involving games was identified, integrating biofeedback from the evidence and training strategies, focusing on the solution proposed, in order to support the execution of the therapy efficiently in the home of young people with cerebral palsy. In [24], a systematic review was presented which analyzed in depth three works associated with lesions due to brain injury: cerebral palsy, stroke, and children with hemiparesis; concern with developing works contributing to the motor recovery of neurological patients was noted.

Additionally, in [18], the contribution of serious games was validated, which were developed for rehabilitation, and it was determined how they can be used as an evaluation tool for the function of the upper limb in patients with advanced Friedreich’s ataxia. This is a hereditary disease of the central nervous system and the peripheral nervous system which causes gait ataxia, dysmetria, dysarthria, dysphagia, severe proprioceptive and superficial sensory loss, weakness, limb atrophy, and loss of muscle tone or spasticity or a combination of both, among other complications related with the senses [57].

Another aspect of interest related to human health is that of energy expenditure in the execution of physical activity, given that energy expenditure and the metabolism substrate are important elements when considering physical activity, and, from their characteristics, it is possible to establish treatments to improve a person’s quality of life [58]. Hence, in [23], through the use of Kinect and Microsoft SDK, an estimation was made of the mechanical work carried out by the body and, thus, it was possible to calculate the metabolic energy using predictive algorithmic models.

Concerning the evaluation and analysis of the range of movement, and the lesions in the upper limbs derived from different clinical conditions, in [7,9,12,16], and in one of the experiments presented in [15], it was identified that the contribution of these technologies to a patient’s motor recovery is positive, as it helps to overcome the limitation of traditional rehabilitation methods.

**Figure 8:**
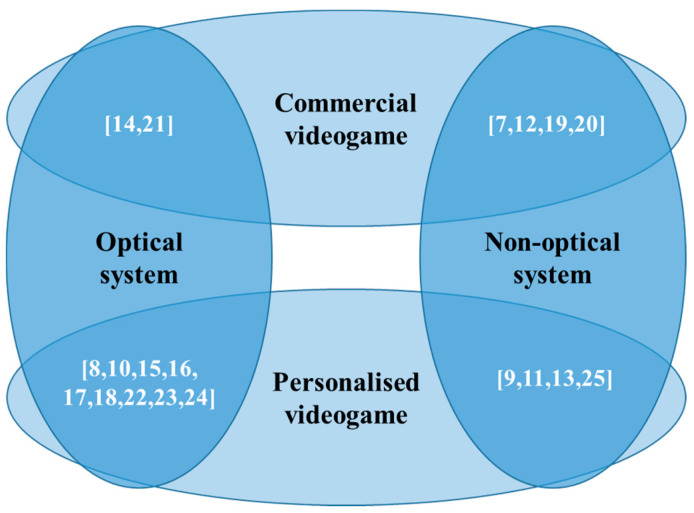
Paper classification according to the technologies used.

In conclusion, the findings of the analysis of the articles which met the inclusion criteria of the review can be classified as is shown in the Venn diagram in Figure 8.

It is necessary to mention that, while optical motion capture systems (11 papers) presented problems with sensor occlusion, in the systems using IMUs or non-optical systems (eight papers), the investigations were left open in order for future research to obtain better precision and to correct the drift generated by the magnetometer. In addition, for the years 2019 and 2020, the literature consulted registered an increase in the use of non-optical motion caption systems (five papers), as opposed to optical systems (two papers).

Furthermore, regarding the use of videogames in physical rehabilitation, there was a clear trend toward the development of personalized videogames (13 papers) and, on fewer occasions, commercial videogames were used (six papers).

Taking into account that the objective of the investigation included the integration of motion capture systems and videogames in upper limb physical rehabilitation, it is appropriate to mention that future investigations could focus on the development of technological tools involving IMUs and the independent development of videogames for the support of said processes.

## 4. Discussion

According to the importance of physical and functional rehabilitation in the quality of life of patients and the people around them, in this review, the technological contributions developed in the past few years in this field were identified, mainly regarding the inclusion of videogames and motion capture systems as support in the motor recovery of the upper limb. In the literature, a wide use of Kinect was identified as the motion capture system, although there were some limits regarding the movements carried out in the depth and occlusal planes of the limbs, i.e., the visual interruption between the camera and some of the body segments, as well as the capture of data in some specific positions (for example, sitting). Furthermore, aspects related to precision were considered in [59,60,61], with greater emphasis when it comes to physical rehabilitation, where precision can be a determining factor in the process. Even so, this sensor was used as a complement in the motor recovery therapies or in works focused on the validation of different attributes such as the usability of the technologies proposed or the verification of motion evaluation methods [10,14,16,17,18,21,22,23]. Among commercial products, not only Microsoft Kinect was used; the use of Nintendo Wii with its Balance Board and the Myo bracelet was reported, allowing validations in the medical field thanks to the fact that they have a lesser cost in comparison with clinical systems, such as Vicon, OptiTrack, and Qualisys, among others.

In this sense, comparisons were made of different motion capture systems with respect to Vicon, OptiTrack, or Qualisys considered to be the gold standard, against which those systems using inertial measurement units have shown a comparable performance [41,42,43,44,45,62] denoting the reliability, accessibility, accuracy, and portability offered by IMUs. In this way, inertial sensors become a good option to be used in the medical field to support motor and functional recovery processes, which require precise measurements with an accessible cost in order to be mass-produced.

Currently, novel motion capture technology involving video alone is available. Using tools from machine learning, researchers have demonstrated that tracking joints of multiple human figures may be achieved [63]. The potential of this approach is enormous, since it would enable implementing games for rehabilitation using hardware available in most dwellings. Nevertheless, for real-time operation, these methods still require powerful graphics hardware, which limits their availability at the moment.

On the other hand, the use of serious videogames has increased due to the lack of motivation of patients when they are in the process of motor recovery. In the face of this, individual, cooperative, and competitive video games have been used. Commercial video games were used in [7,12,19,20,21], which, despite encouraging the execution of physical activity and supporting the player’s motivation, were not adapted to the particular characteristics of physical rehabilitation. For this reason, most of the studies proposed active video games specifically for rehabilitation, to increase motivation and adherence to therapies [8,11,13,15,16,17,18,22,23,25], in some cases associated with a configuration module allowing the health professional to adjust the characteristics of the game according to the diagnosis and progress of the patient in treatment [9,10,14,24]. In the particular case of commercial rehabilitation products, such as ArmeoSenso, Bimeo, or JRS Wave, they respond adequately to such requirements in the area of physical rehabilitation, although the additional costs involved must be taken into account.

When referring to the use of commercial products, i.e., videogames and motion capture systems, it should be noted that they are an important contribution to the field of rehabilitation. However, they are not certified as medical products [15] and, therefore, to include them in a clinical routine, it is recommended that a thorough preliminary study be carried out or, if possible, a design and development procedure, guided by health professionals, to obtain products that respond to the specific needs of the rehabilitation process. Among the particular characteristics of a videogame for rehabilitation, it is worth mentioning that it should have simple visual backgrounds, clinical diagrams in accordance with the patient’s situation, and configurability in terms of range of movement, speed, and recovery time, among other aspects of the process [18].

Although this review included works that used videogames and motion capture systems in physical rehabilitation, not all the works analyzed integrate these components into a single product or system, i.e., the information generated by these technologies was disconnected, making complete and timely analysis difficult in motor recovery therapy.

One of the fundamental aspects in order to achieve the objectives of a physical rehabilitation process is that it is adequately monitored and controlled, and that it is adjustable in a timely manner regardless of whether the patient and the health professional are in the same geographical location or not. For this reason, an optimal system to support physical rehabilitation should integrate various functionalities and technologies, including an accurate and portable motion capture system, as well as a customized active video game module to encourage patient motivation and guide them properly in the execution of therapy. It is also important that the system has a management and monitoring module of the rehabilitation plan assigned to each patient in real time, making it possible to manage the electronic medical record of rehabilitation processes.

In this sense, out of the works included in this review only five presented home rehabilitation systems that allow the therapist to remotely adjust and monitor the configuration of the game according to the patient’s rehabilitation objectives, incorporating the recording of information in an associated computer system. Out of these, in [8], IMUs are used, in [9,13,20] the Kinect sensor was used, and two of the three works analyzed in [23] used the 5DT Data Glove Ultra and the Nintendo Wiimote. Moreover, in four of these five works, videogames developed specifically for rehabilitation were proposed. In this sense, it was identified that this type of system offers a significant contribution to the processes of motor recovery and that it is important that the information gained from the therapies carried out by a patient in a location is convenient for them and registered correctly, such that the process is evaluated in a timely and reliable manner. Thus, telerehabilitation involving a system with these components offers proper support for the management of the process, benefitting patients, their caregivers, and the medical team involved.

## 5. Conclusions

The ability to carry out basic daily activities autonomously is an aspect related to an individual’s quality of life. People can lose their mobility and their capacity to execute daily activities for different reasons, as in the case of neurologic diseases or other clinical conditions. In order to recover functionality, physical rehabilitation systems are implemented that require, in addition to knowledge and orientation from professionals in the area, tools and technologies which provide precision and optimize the process. Motivation and commitment of the patient are also required, as reported in the works analyzed in this research.

This review included studies which support the physical rehabilitation of the upper limb with the use of videogames and motion capture systems, and it identified 19 documents which met the criteria of eligibility defined for this investigation. In the documents analyzed, it was found that, concerning motion capture systems, the use of Microsoft Kinect is prominent, due to its affordability and ease of use. There was also a strong trend regarding the implementation of IMUs given their precision and portability.

Concerning the affordability of the technologies used, it can be stated that most of the works used commercial systems and complemented them with the development of components allowing the adjustment of the technology to rehabilitation processes. Development mainly involved personalized and configurable videogames that respond to some requirements of the motor rehabilitation process, especially attending to the need to foment, increase, and maintain the motivation of the patient in the execution of the therapy. In general, the works showed the advantages provided by the use of active videogames in the recovery of patients, as long as they are designed and developed with the accompaniment of physical and functional rehabilitation professionals, and that they can be used in the patient’s environment.

The studies analyzed included videogames, as well as motion capture systems, although only 26% of these works integrated the different components into one sole product and complemented them with a system that manages the data of the patients for respective monitoring throughout therapy. Thus, in general, this review identified that an optimal system to support physical rehabilitation should include a motion capture system that offers precision and portability, a module of active videogames that are configurable to the particular needs of each patient’s recovery, which permit motivation and proper guidance in the execution of the therapies and, lastly, a computer system which allows the management and monitoring of the rehabilitation plan assigned to each patient, attending to the fundamental aspects of telerehabilitation.

## Figures and Tables

**Table 1:** Search parameters in the different databases.

Database	Search Parameters
Scopus	TITLE-ABS-KEY (((rehabilitation OR health OR “physical therapy” OR “musculoskeletal”) AND (videogames OR “video games” OR “video-games” OR “serious videogames” OR “serious games” OR “serious video games” OR “exergames” OR “exergaming” OR “active videogames”) AND (“upper limb” OR “elbow” OR “shoulder” OR “arm” OR “wrist” OR “humerus”) AND (“inertial sensor” OR “motion capture” OR “motion capture system” OR mocap OR wearable))) AND (LIMIT-TO (PUBYEAR, 2020) OR LIMIT-TO (PUBYEAR, 2019) OR LIMIT-TO (PUBYEAR, 2018) OR LIMIT-TO (PUBYEAR, 2017) OR LIMIT-TO (PUBYEAR, 2016) OR LIMIT-TO (PUBYEAR, 2015))
PubMed	((rehabilitation OR health OR “physical therapy” OR “musculoskeletal”) AND (videogames OR “video games” OR “video-games” OR “serious videogames” OR “serious games” OR “serious video games” OR “exergames” OR “exergaming” OR “active videogames”) AND (“upper limb” OR “elbow” OR “shoulder” OR “arm” OR “wrist” OR “humerus”) AND (“inertial sensor” OR “motion capture” OR “motion capture system” OR mocap OR wearable))
IEEE Xplore and Web of Science	((rehabilitation OR health OR “physical AND therapy” OR musculoskeletal) AND (videogames OR “video AND games” OR video-games OR “serious AND videogames” OR “serious AND games” OR “serious AND video AND games” OR exergames OR exergaming OR “active AND videogames”) AND (“upper AND limb” OR “elbow” OR “shoulder” OR “arm” OR “wrist” OR “humerus”) AND (“inertial AND sensor” OR “motion AND capture” OR “mocap” OR “motion AND capture AND system” OR wearable))

**Table 2:** Search parameters in the different databases. IMU, inertial measurement unit; MS, Microsoft; ROM, range of motion; N/A, not applicable.

No.	Mocap System	Clinical Condition	Population (Sample) *	Technology Used **	Part of the Body Rehabilitated	Reference
1	IMU	Cerebral palsy	19 P	Mixed: Myo bracelet, adapted commercial videogame (Dashy Square and personalized software development)	Hand and wrist	[7]
2	MS HoloLens	ROM	25 H	Mixed: MS HoloLens and developed videogame	Shoulder	[8]
3	IMU	Stroke	8 H	Proposed system: an environment of games and software for the therapist	Upper and lower limbs	[9]
4	MS Kinect	Upper limb lesions	10 P	Mixed: MS Kinect V2, videogame development, and web application	Arm	[10]
5	IMU	N/A	11 H	Proposed system	Arm	[11]
6	IMU	N/A	N/A	Commercial: ArmeoSenso	N/A	[12]
7	IMU	Upper limb lesions	10 P	Mixed: Myo bracelet and a developed videogame	Arm	[13]
8	MS Kinect	Stroke	30 H	Commercial: MS Kinect V2 and Mystic Isle (videogame integrated to Kinect)	Upper part of the human body	[14]
9	MS Kinect	Stroke	11 P	Mixed: MS Kinect and a developed videogame	Arm	[15]
10	MS Kinect	Stroke	24 P	Mixed: MS Kinect and Recovery Rapids ™ (personalized videogame)	Arm	[16]
11	MS Kinect	ROM	10 H	Mixed: MS Kinect and development of a personalized system	Arm	[17]
12	MS Kinect	Friedreich’s ataxia	27 P, 43 H	Mixed: MS Kinect and development of a videogame.	Arm	[18]
13	IMU	Stroke	29 P	Commercial: Bimeo	Arm	[19]
14	IMU	Stroke	11 P	Commercial: ArmeoSenso.	Arm	[20]
15	MS Kinect	Stroke	74 P	Commercial: JRS Wave	Human body	[21]
16	MS Kinect	Stroke	18 P, 12 H	Proposed system	Upper part of the human body	[22]
17	MS Kinect	Energy expenditure	19 H	Mixed: MS Kinect and development of a system	Human body	[23]
18	Other optical systems	Lesions due to brain injury	N/A	Mixed	Hand	[24]
19	Orthosis with IMU	Stroke	7 P	Proposed system	Wrist and hand	[25]

* Population: P = patients; H = healthy participants. ** Technology used: commercial and/or developed.
